# Neural tube derived Wnt signals cooperate with FGF signaling in the formation and differentiation of the trigeminal placodes

**DOI:** 10.1186/1749-8104-3-35

**Published:** 2008-12-15

**Authors:** Claire A Canning, Lily Lee, Sarah Xinwei Luo, Anthony Graham, C Michael Jones

**Affiliations:** 1 Institute of Medical Biology, A*STAR, 8A Biomedical Grove, #06-06 Immunos, Singapore 138648, Republic of Singapore; 2 MRC Centre for Developmental Neurobiology, Kings College London, Guys Campus, London, SE1 1UL, UK

## Abstract

**Background:**

Neurogenic placodes are focal thickenings of the embryonic ectoderm that form in the vertebrate head. It is within these structures that the precursors of the majority of the sensory neurons of the cranial ganglia are specified. The trigeminal placodes, the ophthalmic and maxillomandibular, form close to the midbrain-hindbrain boundary and many lines of evidence have shown that signals emanating from this level of the neuraxis are important for the development of the ophthalmic placode.

**Results:**

Here, we provide the first evidence that both the ophthalmic and maxillomandibular placodes form under the influence of isthmic Wnt and FGF signals. Activated Wnt signals direct development of the *Pax3 *expressing ophthalmic placodal field and induce premature differentiation of both the ophthalmic and the maxillomandibular placodes. Similarly, overexpression of *Fgf8 *directs premature differentiation of the trigeminal placodes. Wnt signals require FGF receptor activity to initiate *Pax3 *expression and, subsequently, the expression of neural markers, such as *Brn3a*, within the cranial ectoderm. Furthermore, fibroblast growth factor signaling via the mitogen activated protein kinase pathway is required to maintain early neuronal differentiation within the trigeminal placodes.

**Conclusion:**

We demonstrate the identity of inductive signals that are necessary for trigeminal ganglion formation. This is the first report that describes how isthmic derived Wnt signals act in concert with fibroblast growth factor signaling. Together, both are necessary and sufficient for the establishment and differentiation of the ophthalmic and maxillomandibular placodes and, consequently, the trigeminal ganglion.

## Background

The sensory neurons of the head develop differently from those of the trunk. In the trunk, these neurons are derived exclusively from the neural crest, while in the head, sensory neurons are generated from neural crest and focal thickenings of the embryonic ectoderm, the neurogenic placodes [1,2]. A ganglion in which this complexity is readily observed is the trigeminal. The neurons of this ganglion arise from two distinct placodes, the ophthalmic and the maxillomandibular, which form alongside the midbrain-hindbrain junction. They also have a neural crest cell component [1,3,4]. Trigeminal neurons are generated initially by the ophthalmic placode and, subsequently, by the maxillomandibular placode. The neural crest derived neurons are generated at significantly later stages. The trigeminal ganglion is of great importance. It conveys somatosensory information from the face, but there is relatively little understanding of how its early development is controlled, particularly with respect to the development of the ophthalmic and maxillomandibular placodes.

The ophthalmic placode forms early, at the six somite stage in chick, and is marked by the robust expression of *Pax3 *[5]. This placode lies adjacent to the midbrain-hindbrain boundary and evidence suggests that its development is under the influence of signals emanating from the midbrain. The placodal cells extend anterior-laterally in the ectoderm and then delaminate into the underlying mesenchyme as neuronal differentiation begins. The maxillomandibular placode forms slightly later, by stage 12 in chick [6].

An in-depth analysis was carried out to address the competence and specification of *Pax3 *expression in relation to the ophthalmic trigeminal placode [7]. The findings are summarized as follows. At the three somite stage, head ectoderm rostral to the first somite is competent to express *Pax3 *when grafted into cranial ectoderm, as shown by quail-chick transplantations. Different regions, comprising forebrain level, midbrain level, rhombomere 2–3 and otic level quail ectoderm exhibited different levels of competence when grafted into the midbrain region of chick hosts. Competence to express *Pax3 *was highest in anterior cranial ectoderm (forebrain, midbrain and rhombomere 2–3). Previous work has outlined a role for neural tube-ectoderm interactions in relation to *Pax3 *expression [5], and Baker *et al*. [7], in explant studies, confirmed that this interaction was direct. Foil barriers implanted between the neural folds and adjacent ectoderm, from two to nine somites, lead to a complete loss of *Pax3 *expression in the developing ophthalmic placode. The loss of *Pax3 *expression was coupled with a subsequent loss of developing neurons. Inductive cues from the neuroepithelium were proposed, therefore, to be involved in controlling ectodermal *Pax3 *expression. Lassiter *et al*. [8] undertook further studies to describe the role of canonical Wnt signaling within the cranial ectoderm itself and the role this signaling pathway plays in maintaining placodal fate. Furthermore, an RT-PCR screen was recently carried out to identify candidate secreted factors that might play a role in trigeminal placode development [9]. This particular strategy involved screening for receptors expressed directly within the cranial ectoderm, including those of the fibroblast growth factor (FGF) and Wnt family, some of which have been previously characterized [5].

However, the search for neural tube derived signals regulating the development and differentiation of the trigeminal ganglion is ongoing. Here we present evidence that Wnt and FGF signaling are both necessary and sufficient to regulate the earliest ophthalmic placode marker, *Pax3*. Neural tube derived Wnt signals have a temporal requirement in the establishment of the early trigeminal placode but are no longer required once the *Pax3 *domain is specified. Ectopic activation of Wnt1 or FGF8 at the midbrain-hindbrain boundary is sufficient to direct differentiation of both the ophthalmic and maxillomandibular branches of the trigeminal ganglion. Under their influences, precursor pools are increased and neuronal differentiation occurs prematurely, albeit normally. Additionally, we describe how FGF signaling, via the mitogen-activated protein kinase (MAPK) pathway, maintains further differentiation of the developing placodal branches. This is the first instance whereby neural tube derived Wnt signals in conjunction with FGF signaling have been shown to influence the establishment and differentiation of the trigeminal ganglion.

## Materials and methods

### Electroporation of DNA constructs

Fertilized White Leghorn eggs were incubated at 38°C in a humid atmosphere. pCAGGSmWnt1 (2 μg/μl) [10], pCAGGSmFgf8b (2 μg/μl) [11] or dominant negative Wnt1(2 μg/μl) [12] were electroporated together with pCAGGSeGFP (1 μg/μl) using an IntracelTSS20 at the following settings; 20 volts, 4 pulses, 50 millisecond duration, space 950 milliseconds. At the five to six somite stage, DNA was targeted to the neural tube at the presumptive midbrain region, and at HH10 (Hamburger and Hamilton stage 10), DNA was targeted to the neural tube, encompassing midbrain and anterior hindbrain.

### *In situ *hybridization and immunohistochemistry

Wholemount *in situ *hybridization using digoxygenin labeled probes was performed according to [13]. Double *in situ *hybridization using digoxygenin and fluorescein (FITC) labeled probes was carried out as previously described [14]. Embryos were flat mounted as previously described [15]. Following *in situ *hybridization, embryos were paraffin embedded and processed to generate 20 μm sections using a microtome (Leica). For antibody staining, embryos were fixed in 4% paraformaldehyde overnight, followed by dehydration and subsequent rehydration through a methanol:PBSTx (phosphate-buffered saline plus 1% Triton X-100, 1% serum) series and blocked for 1 hour in PBSTx. The following antibodies were used: anti-neurofilament (RMO-270, Zymed, San Francisco, USA,; 1:300), anti-Islet 1 (clone 39.4D5, Developmental Studies Hybridoma Bank, DSHB, University of Iowa, Iowa City, USA; 1/10), anti-HNK1 (clone VC1-1, Sigma, MI, USA; 1/500), anti-double phosphorylated Erk1/2 (dpErk1/2, Cell Signaling, Boston, MA, USA; 1/200) and anti-green fluorescent protein (anti-GFP; Calbiochem, an affiliate of Merck, Germany,; 1/500). Embryos were washed in PBSTx five to six times, blocked again in PBSTx and incubated with appropriately species matched horseradish peroxidase (Dako, Glostrup, Denmark; 1:400), Alexa^488^, or Alexa^594 ^conjugated (Molecular Probes/Invitrogen, CA, USA) secondary antibodies. All antibody incubations were carried out at 4°C overnight. horseradish peroxidase detection was performed using DAB tablets (Sigma). Confocal analysis was performed using a Zeiss LSM 510 Meta confocal microscope, and images were processed using Image J software (NIH). Embryos were fixed in 4% paraformaldehyde then incubated overnight in 30% sucrose and embedded in optimal cutting temperature (OCT) compound. Cryostat cut sections (16 μm) were blocked and incubated with antibodies as above.

### Western blot analysis

Proteins from explants were extracted with modified RIPA buffer (20 mM Tris, pH 8.0, 1 mM EDTA, 0.1% NP-40, 10% glycerol, 1 mM Na_3_VO_4_, 1 mM PMSF, 1× Proteinase inhibitor cocktail) (Roche). Samples were separated by SDS-PAGE on an 8% bis-acrylamide gel and transferred to PVDF membrane (Hybond, GE Healthcare, NJ, USA). The membrane was blocked in 5% skimmed milk and incubated overnight in 1× TBS/0.1% Tween 20/5% milk containing a dilution of double phosphorylated Erk1/2 (dpErk1/2; 1/2000; Cell Signalling), or nuclear specific dephosphorylated *β*-catenin antibodies (1/500; Calbiochem). Blots were then stripped (stripping buffer: 2% SDS, 100 mM *β*-mercaptoethanol, 50 mM Tris, pH 6.8) and probed with anti-Actin (1/2000; Chemicon), which served as a loading control. Peroxidase conjugated secondary antibodies (1/1000) were detected using chemiluminescence (ECL+, Amersham).

### Collagen explant assays

Explant tissues were isolated using sharp tungsten needles to remove cranial ectoderm and underlying mesenchyme from surrounding neural tissue at the five to six somite and ten somite stages. Cranial ectoderm was isolated at the level of the presumptive midbrain to hindbrain level (five to six somites) or at the midbrain to rhombomere 2 level (HH10). Collagen solution was made by adding 100 μl of 10× MEM (Sigma) and 100 μl bicarbonate buffer (0.1 M NaOH, 240 mM NaHCO3) to 0.8 ml collagen (PurCol, Nutacon, The Netherlands). Explants were cultured alone in 75% (v/v) Optimem: 25% (v/v) Leibovitz medium (Invitrogen), or supplemented with PD184352 (5 μM; a gift from Professor Philip Cohen, University of Dundee), SU5402 (50 μM; Calbiochem), 0.5 μg/ml Wnt3A (R and D Systems, Minneapolis, MN, USA), 0.1 μg/ml FGF8b (R and D Systems) or 0.5 μg/ml SFRP2 (R and D Systems). In each experimental setup, all explants used for an individual figure were cultured and processed simultaneously such that experimental conditions were comparable.

### Axon outgrowth assay

Cranial ectoderm explants were dissected at HH10 and grown for 48 hours on cover slips that were coated with 0.1% poly L Lysine followed by 5 μg/ml laminin. Explants were either grown in recombinant Wnt3A plus FGF8b, or grown alone [16]. Explants were subsequently fixed and processed for anti-neurofilament staining as above.

## Results

### Elevated Wnt signals from within the neural tube result in increased numbers of *Pax3 *positive cells within the ophthalmic branch of the trigeminal placode

**Figure 1:**
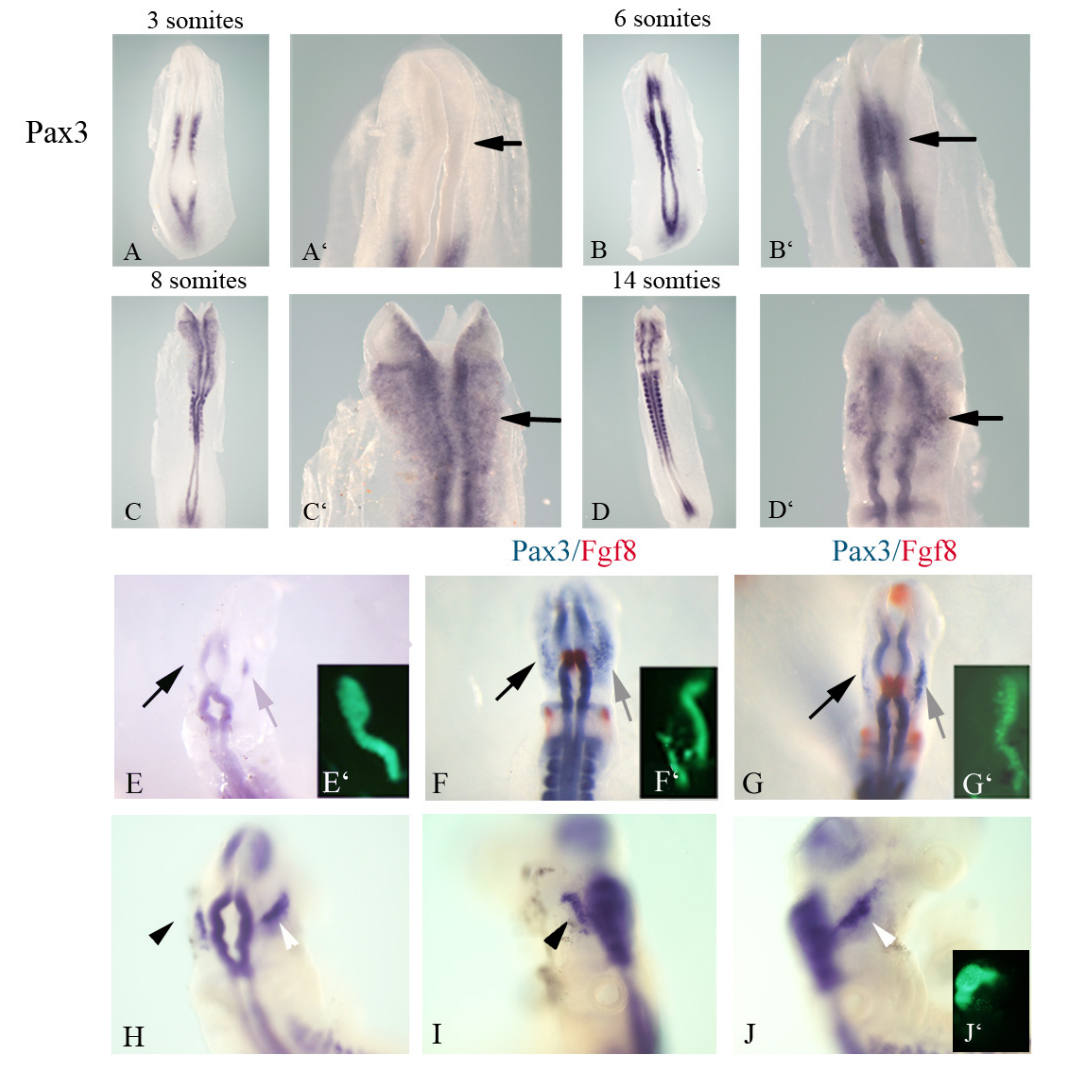
**Activation of the Wnt pathway results in increased *Pax3* expression in the early ophthalmic trigeminal placode.** In situ hybridization showing *Pax3* (blue) (A-J) and *Fgf8* (red) (F, G). (A-D) Expression of *Pax3*; (A'-D') are higher magnification images of embryos in (A-D). At 3 somites, *Pax3* expression is detectable in the somitic mesoderm, but no transcripts are detected in the neural folds or adjacent ectoderm (A, A', black arrow). At 6 somites, *Pax3* transcripts are detectable in the neural folds (B, B'), and transcripts begin to emerge in the cranial ectoderm (black arrow). *Pax3* expression becomes more abundant by 8 somites (C, C', black arrow). At 14 somites (HH11+) the *Pax3* expressing placode is evident (D, D', black arrow. (E-J) CAGGS Wnt and green fluorescent protein (GFP) are co-electroporated at the midbrain level of the neural folds and targeted to the right side. GFP expression is shown in insets (E', F', G', J'). Embryos were cultured for 6 hours (E, F, G) or 12 hours (H-J). (E) Overexpression of Wnt1 at 6 somites leads to a premature appearance of the earliest *Pax3* positive presumptive ophthalmic placodal cells (grey arrow). Embryos injected at the 8 (F) or 10 somite stage (G) also yield a greater number of *Pax3* positive cells in the adjacent ectoderm (F, G, compare grey arrow, right, with black arrow, left). No change in isthmic *Fgf8* expression (red) is observed (F, G). (H) HH10 embryo electroporated and incubated for 12 hours. The white arrowhead (H) delineates the expanded *Pax3* expressing domain compared to the uninjected side (black arrow head). (I, J) Left and right lateral views, respectively, of the embryo viewed dorsally in (H). The arrowheads point to the ophthalmic branch of the trigeminal placode on the injected side (J, white arrowhead), and uninjected side (I, black arrowhead).

We have previously demonstrated the necessity for a sustained interaction between isthmic Wnt and FGF signaling to maintain *Fgf8 *expression within rhombomere 1 [14]. Here we describe how these signaling molecules, while regulating neural patterning, also direct overlapping and discrete aspects of trigeminal placode development and subsequent neuronal differentiation. *Pax3 *is the earliest detectable marker of the ophthalmic placode. We undertook a time course analysis in the early chick embryo to address precisely when the earliest *Pax3 *positive cells can be detected within the cranial ectoderm (Figure 1A–D). This expression analysis was critical to determine at what stages gain and loss of function experiments should be carried out *in ovo*, and for explant analysis (discussed below). The emerging ophthalmic placode can be visualized from HH8+ (6 somites) onward in the chick embryo by the expression of *Pax3 *(Figure 1B and arrow in 1B') and is robustly detectable by the 8 somite stage (Figure 1C and arrow in 1C') [5]. The appearance of Pax3 protein is detected in the cranial ectoderm adjacent to the midbrain-hindbrain boundary at the seven somite stage [7].

To determine if the earliest *Pax3 *positive placodal cells respond to emerging isthmic signals, Wnt1 was overexpressed in the neuroepithelium of 6, 8 and 10 somite embryos and incubated for 6 or 16 hours. Overexpression of Wnt1 in the neural tube at the 6 somite stage, when *Pax3 *expression begins to be detected in the adjacent ectoderm, resulted in increased *Pax3 *expression corresponding to the early ophthalmic placode (Figure 1E; n = 8/8). Similar results were observed when Wnt1 was overexpressed at the 8 or 10 somite stage, when *Pax3 *positive cells are specified to a placodal fate. When these embryos were incubated for 6 hours, until they reached 12 and 14 somites, respectively, the *Pax3 *expressing domain within the ophthalmic placode was larger (n = 7/8, 8 somites; n = 10/10, 10 somites; Figure 1F, G, respectively). Notably, the isthmic *Fgf8 *domain had not yet responded to activated Wnt signaling by this time point; we could only detect ectopic expression of *Fgf8 *9 hours post-electroporation of Wnt1 (CAC and CMJ, unpublished results). These results suggest that the earliest expansion of the trigeminal placode may be responding to Wnt signals alone, although we do not rule out the role of related FGF and Wnt family members also expressed within the midbrain-hindbrain region. Overexpression of Wnt1 at HH10, a time when the *Pax3 *domain is committed to placodal fate, expands the population of *Pax3 *positive precursor cells within the ophthalmic branch, when analyzed at HH15 (Figure 1H, J). These results demonstrate that overexpression of Wnt1 within the midbrain over a significant period of time – from the time the earliest *Pax3 *positive cells emerge until they are committed to form the placode-results in an increased domain of *Pax3 *expression in the cranial ectoderm. Therefore, Wnt signals are involved in the early development of the trigeminal placodes and appear to act simply by expanding the number of cells expressing the earliest known marker, *Pax3*. Wnt signals are also involved in neural crest migration. However, overexpression of Wnt1 at HH7 (Additional file 1) and concomitant DiI labeling of the neural folds resulted in an expanded *Pax3 *expression domain that is not overlapping with labeled migrating crest.

### Attenuating midbrain Wnt signals results in a reduction of the number of *Pax3 *positive cells in the emerging ophthalmic placode

**Figure 2:**
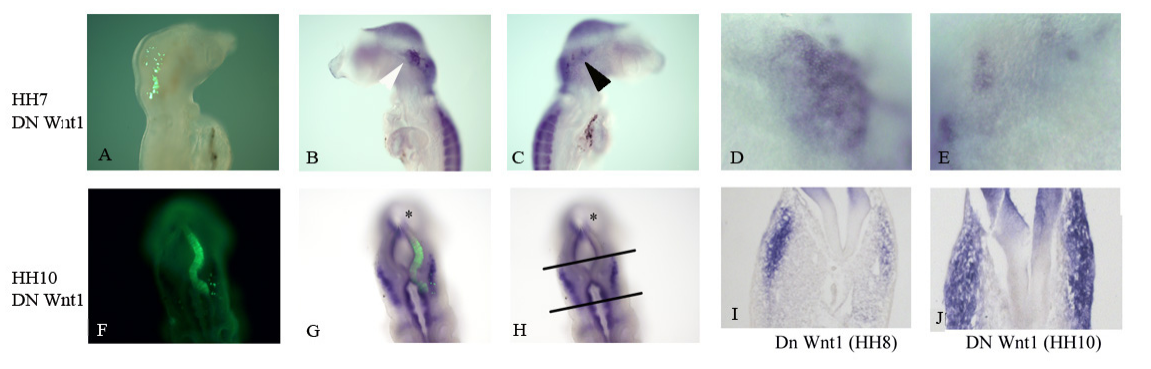
**Isthmic derived Wnt signals are required at HH8 for maintenance of the early ophthalmic placode identity.*** In situ *hybridization for *Pax3 *(blue) (B-E, G-J). GFP expression is shown in green (A, F and G). A dominant negative form of Wnt1 (DN Wnt1) was targeted to the right-hand side of the neural tube in all cases. Embryos were co-electroporated with GFP at HH7 (A) or HH10 (F, G) and analyzed 16 and 12 hours later until they reached approximately HH13. When Wnt ligands are perturbed at HH8, expression of *Pax3 *is reduced in the placodal ectoderm (C, black arrow head; E). The white arrowhead (B) indicates the normally developing placode on the un-injected side. When DN Wnt1 is injected at HH10 *Pax3 *expression in the placodes is not altered when analyzed at HH13 (G, H, J). The lines in (H) mark the extent through which *Pax3 *is expressed in the ophthalmic placodes on both sides. Embryos injected with dominant negative Wnt1 at HH8 (I) or HH10 (J) were sectioned to reveal the localization of *Pax3 *expression. Less *Pax3 *positive cells were observed in general, and within the mesenchyme, when dominant negative Wnt1 was injected at HH8 (I, right hand side). No obvious differences were observed when dominant negative Wnt1 was electroporated after HH10 (J). Asterisks delineate midbrain (G, H).

Previous results have reported that blocking the canonical Wnt pathway within the cranial ectoderm results in cells failing to maintain trigeminal placode fate [8]. We have confirmed these results and observe that both *Pax3 *and, consequently, *Islet1 *expression are diminished when a dominant negative form of *β*-catenin is expressed directly in the cranial ectoderm (data not shown). Furthermore, these authors concluded from gain of function studies that although Wnt signaling within the ophthalmic placode was necessary, it was not sufficient for ophthalmic placode induction. Overexpression of constitutively active *β*-catenin did not result in ectopic expression of *Pax3 *positive cells. However, gain of function studies outlined above demonstrated that elevated Wnt1 signals within the midbrain-hindbrain region induced development of the ophthalmic placode and expanded the expression of the earliest marker, *Pax3*. To address whether Wnt signals emanating from the neural tube were required for the correct development of the ophthalmic placode, we electroporated a dominant negative form of Wnt1 from the time we could first detect *Pax3 *expression. CAGGS GFP was co-electroporated directly into the neural tube and DNA for both constructs was targeted to the right hand side. Attenuation of Wnt signals at HH8 resulted in fewer *Pax3 *positive cells within the early placode when analyzed at HH13 (Figure 2C, E; n = 15/18). Coronal sections revealed a reduction in the number of *Pax3 *positive cells as they begin to delaminate into the mesenchyme (Figure 2I). However, when dominant negative Wnt1 was introduced into the neural tube after HH10, no change in placodal *Pax3 *expression was observed (Figure 2F–H, J; n = 15/16). Notably, a reduction of isthmic *Fgf8 *expression [14] and a loss of *Pax3 *transcripts within the neural tube was observed (data not shown), indicating that both dominant negative constructs were functional. Taken together, these results suggest a temporal requirement for neural tube derived Wnt signals to maintain the early development of the *Pax3 *positive placodal population. This activity appears to be no longer required beyond HH10-12.

**Figure 3:**
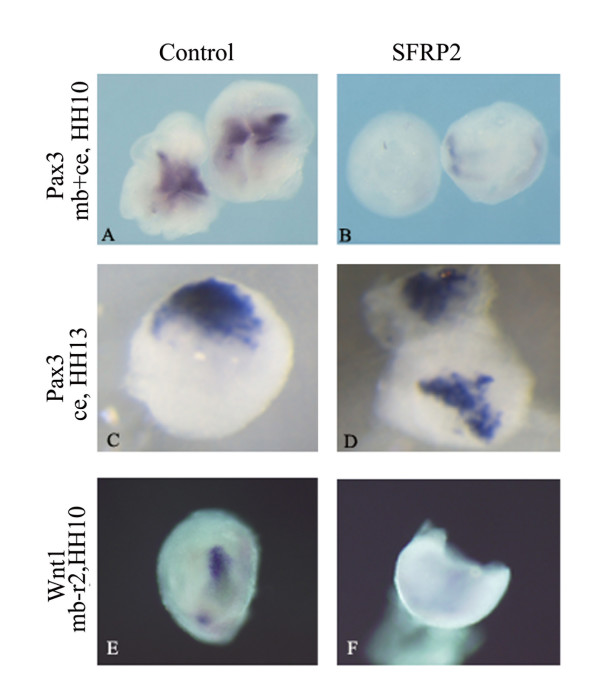
**Wnt signals act in a temporal manner to regulate *Pax3 *expression. Explants isolated at HH10 and HH13 were cultured alone (A, C, E) or with SFRP2 (B, D, F) overnight and stained for *Pax3 *(A-D) and *Wnt1 *(E-F).**  Explants cultured with SFRP2 lost the expression of *Pax3 *(B), compared to controls (A). Cranial ectoderm (ce) isolated at HH13 (C, D) and cultured in the presence of SFRP2 (D) did not lose *Islet1 *expression compared to controls (C). Midbrain to rhombomere 2 (mb-r2) explants express *Wnt1 *when cultured alone (E) but lose endogenous *Wnt1 *transcripts when cultured in the presence of SFRP2 (F) (n = 4/4)

To further address the requirement of Wnt signals post-HH10-12, explants were excised at different developmental time points and cultured in the presence of the Wnt antagonist secreted frizzled-related protein (SFRP)2. Neuroectoderm plus cranial ectoderm was excised at the 8 somite stage and incubated alone (Figure 3A) or in the presence of 0.5 μg/ml of SFRP2 overnight. In the presence of the Wnt antagonist, *Pax3 *expression was lost completely (Figure 3B), compared to control explants (Figure 3A). When ectodermal explants were excised at HH13, the presence of SFRP2 no longer inhibited *Pax3 *expression (Figure 3D). Expression was comparable to that detected when explants were cultured alone (Figure 3C). The same result was observed when ectodermal explants were cultured with adjacent midbrain tissue intact (data not shown). The results from *in ovo *and *in vivo *assays suggest that while Wnt signals are necessary for the onset of *Pax3 *expression, they are dispensable after HH13. We also examined how antagonizing Wnt signals affects the expression of the midbrain *Wnt1*. Midbrain to rhombomere 2 explants grown in the presence of SFRP2 lost *Wnt1 *expression (Figure 3F) compared to controls (Figure 3E). This demonstrates that Wnt signaling serves to regulate the expression of Wnt1 ligands within the neuroepithelium.

### Wnt activity is dependent on FGF signaling to initiate *Pax3 *expression within the cranial ectoderm, and for subsequent neuronal differentiation

**Figure 4:**
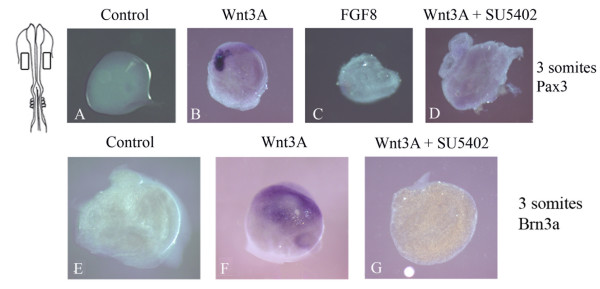
**Wnt signals are dependent on FGF signaling for the onset of *Pax3 *expression and subsequent neuronal differentiation in the cranial ectoderm.**  Cranial ectoderm explants isolated at the 3 somite stage (as depicted in the cartoon diagram) were grown in collagen gel cultures in the presence of Wnt3A (B, F), FGF8 (C), or Wnt3A and SU5402 (D, G). Explants were stained for the expression of *Pax3 *(A-D) and *Brn3a *(E-G). The presence of Wnt3A (B) but not FGF8b (C) was sufficient to induce the onset of *Pax3 *expression. However, Wnt3A is dependent on FGF signals to initiate *Pax3 *expression (D). When cranial ectoderm was isolated and cultured for 24 hours until approximately HH16, Wnt3A (F) resulted in expression of *Brn3a*. Similarly, the activity of Wnt3A was dependent on FGF signals to turn on *Brn3a *expression (G). Control explants cultured overnight were negative for the expression of *Brn3a *(E).

In an attempt to uncouple the activities of isthmic organizer molecules (that is, Wnt1 and FGF8), which is not possible in our electroporation strategies due to their reciprocal feed forward interactions [14], we undertook explant assays to address the roles of these signaling molecules independently. Our *in vivo *analyses, through overexpression of Wnt1 in the neuroectoderm at the onset of *Pax3 *placodal expression, and loss of function studies through perturbation of Wnt signals, suggested that Wnt activity may act alone to induce *Pax3 *expression in the ophthalmic placode. To test this directly, we isolated cranial ectoderm explants from the presumptive midbrain to anterior hindbrain level at the 3 somite stage, before the onset of *Pax3 *expression, and asked whether Wnt or FGF induced *Pax3 *expression. These explants are also isolated before neural crest has migrated. The presence of recombinant Wnt3A protein (a related Wnt1 family member known to activate the canonical Wnt pathway; Figure 4B; n = 8/8) but not FGF8b (Figure 4C; n = 11/11) was sufficient to induce *Pax3 *expression. In control explant cultures, tissue isolated from 3 somite stage embryos did not express *Pax3 *after overnight culture (Figure 4A; n = 19/19). However, given that Wnt and FGF signals act reciprocally in a signaling loop within the neuroepithelium, we addressed whether Wnt activation of *Pax3 *expression was dependent on FGF signaling. In the presence of SU5402 (an FGF receptor antagonist) Wnt3A no longer initiated *Pax3 *expression in ectoderm explants (Figure 4D; n = 8/8). Positive controls for the activity of Wnt3A and FGF8b are shown in Additional file 2. In both cases, isthmic expression of *Fgf8 *was expanded, as previously reported [14]. Given that Wnt activity appeared dependent on FGF receptor activation to initiate *Pax3 *expression, we investigated whether the same holds true for the expression of the neuronal marker *Brn3a*. Similarly, Wnt activity was dependent on FGF signaling to initiate *Brn3a *expression (Figure 4F, G). These results demonstrate that Wnt and FGF signals act together to initiate *Pax3 *expression and, as a consequence, the expression of neuronal markers such as *Brn3a*. Perhaps isthmic organizer activity can not be uncoupled to direct an expansion of placodal *Pax3 *expression *in vitro*. This may account for the insufficiency of constitutively active *β*-catenin, when overexpressed in the cranial ectoderm, to expand *Pax3 *expression, as reported by Lassiter *et al*. [8]. An alternative explanation is that Wnt activity may act independently of *β*-catenin and could directly interact with components of the FGF pathway.

### Overexpression of Wnt1 and ectopic activation of isthmic *Fgf8 *expression results in a hyperdifferentiated trigeminal ganglion

**Figure 5:**
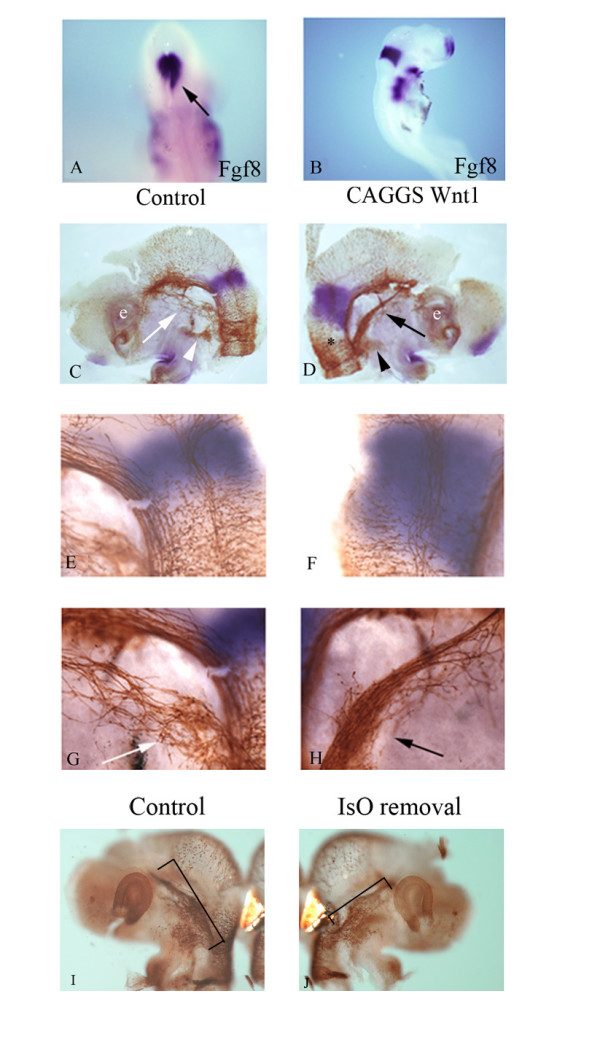
**Overexpression of Wnt1 leads to ectopic activation of *Fgf8 *within rhombomere 1 and a hyperdifferentiated trigeminal.*** Fgf8 *expression (blue) detected by *in situ *hybridization (A-H) and anti-neurofilament (brown) (C-J). Overexpression of Wnt1 at the right-hand side of the neural tube at HH10 results in an expansion of isthmic *Fgf8 *expression, as analyzed at HH16 (A, B, D, F). (A) Dorsal view (anterior is to the top) of the anterior hindbrain showing expansion of *Fgf8 *expression in the right neural tube (black arrow). (B) Embryo as in (A) viewed laterally from the right-hand side. Embryos (C-J) are flat mount preparations, anterior is left (C, E, G, I) and right (D, F, H, J). (C) Bisected head at HH16 showing the normal development of the ophthalmic (C, G, white arrows) and maxillomandibular (C, white arrow head) lobes. (D, H) Elevated Wnt signals and expansion of isthmic *Fgf8 *expression promotes differentiation of both the ophthalmic (black arrows) and maxillomandibular (black arrowhead) branches. When the isthmic organizer (IsO) region is removed (J) and replaced with foil, the trigeminal ganglion appears smaller than the unoperated side (I). (E, F) High magnification images of isthmic domain (C, D). (G, H) High magnification images of ophthalmic lobe in (C, D). The asterisk in (D) delineates the rhombomere 1/2 boundary. The eye (e) is marked for reference in (C, D).

To address later effects of Wnt signals on trigeminal differentiation, Wnt1 was targeted by electroporation to the right side of the neural tube at HH10, and embryos were cultured overnight until they reached HH16. Overexpression of Wnt1 resulted in a caudal expansion of isthmic *Fgf8 *expression (Figure 5A, B, D, F; n = 18/20) as expected [14]. Coincident with expanded *Fgf8 *expression, which was almost double in size (Figure 5F compared to Figure 5E), the ophthalmic trigeminal lobe appeared more differentiated (Figure 5D, H). These results indicate that elevated levels of Wnt1 and ectopic expression of *Fgf8 *from within the neural tube affects differentiation of the trigeminal ganglion. These results point to a growth effect on the trigeminal ganglion as we never observed aberrant targeting or ectopic branching. In an attempt to ablate some neural tube derived signals, the isthmic organizer region and posterior midbrain was dissected away and a foil barrier was inserted to prevent isthmic regeneration. The trigeminal ganglion was visualized using a neurofilament antibody, and on the side where the isthmus was ablated, the ganglion appeared smaller (Figure 5J; n = 6/6) compared to the un-operated side (Figure 5I). These results demonstrate that isthmic derived signals are involved in the maintenance of trigeminal differentiation.

### Elevated Fgf8 signals result in premature differentiation of both the ophthalmic and maxillomandibular branches of the trigeminal placode

**Figure 6:**
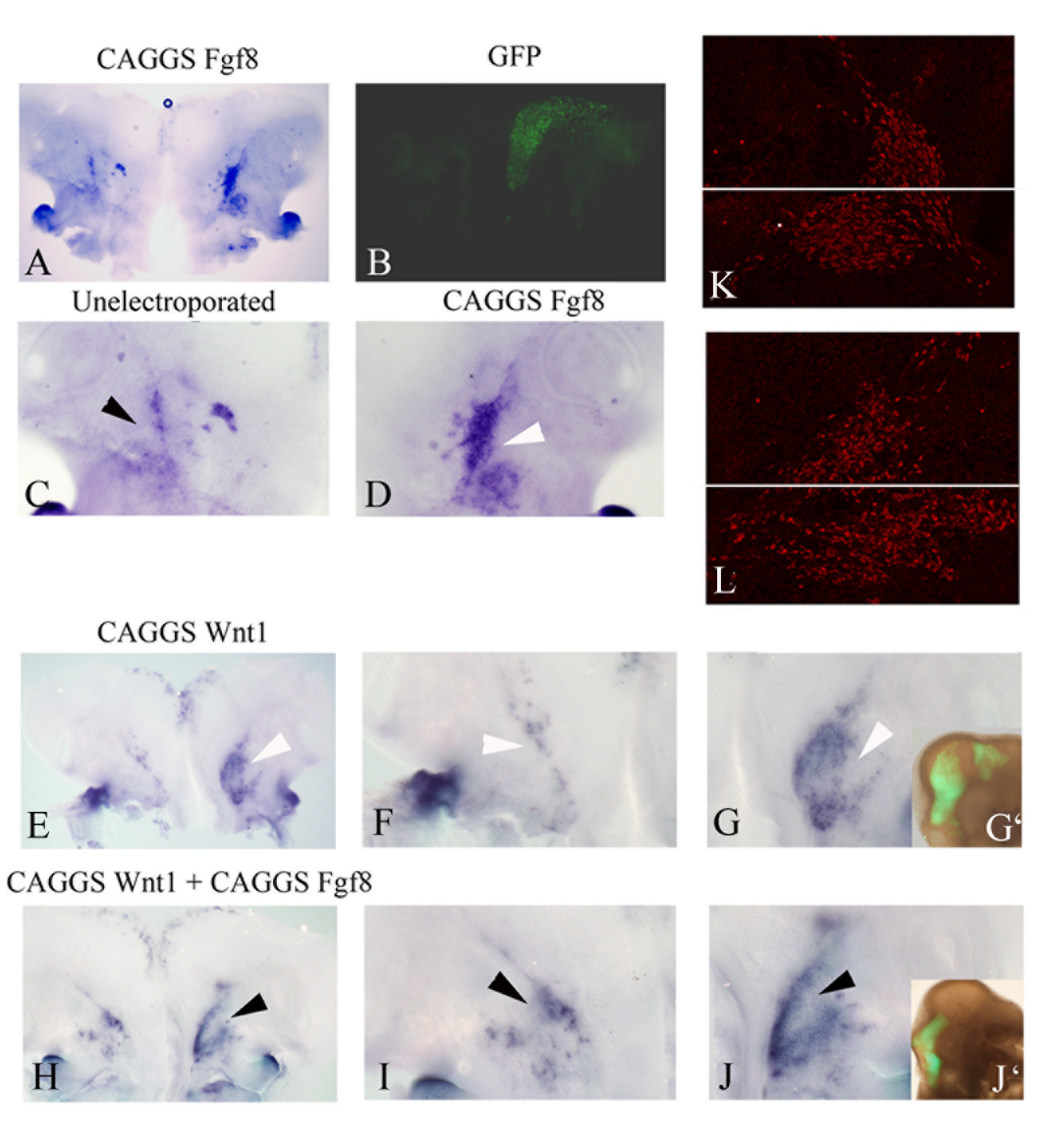
**The ophthalmic and maxillomandibular branches of the trigeminal nerve differentiate in response to activated fibroblast growth factor (FGF) signals within the neural tube.*** In situ *hybridization detects *Islet1 *expression (A, C-D, E-J), green fluorescent protein (GFP) expression (B, G', J') and anti-Islet1 (K, L) expression. FGF8, Wnt1 or FGF8 and Wnt1 were electroporated at the right side of the neural tube at HH10, and embryos were cultured until HH16 (A-L). Overexpression of FGF8 results in premature differentiation of the ophthalmic and maxillomandibular branches (compare white arrow head in (D) with the black arrow head in (C)). Similarly, following electroporation of Wnt1 in the neural tube, the expression of *Islet1 *is more pronounced in the right trigeminal placodes (E, G, white arrowheads) compared to the untargeted side (F, white arrowhead). Overexpression of both Wnt1 and FGF8 together (H, I, J, black arrowheads) also results in similar expansion of *Islet1 *expression. Confocal analysis for *Islet1 *expression (red) within the developing placodes reveals that both the ophthalmic and maxillomandibular branches appear prematurely differentiated (L) compared to the un-targeted side (K). (C, D) High magnification images of flat mount in (A); (F, G) high magnification images of flat mount in (E); (I, J) higher magnification images of flat mount in (H).

To determine whether the activation of isthmic FGF signals can also regulate differentiation of the trigeminal placodes, we electroporated CAGGS FGF8 on the right side of the neural tube at HH10 and examined the expression of a neuronal marker, *Islet1*. When embryos were incubated for 16 hours, until HH16, 100% of the embryos displayed a robust expansion of isthmic *Fgf8 *and *Wnt1 *expression (Additional file 2F, G, J, K; n = 8/8). Overexpression of *Fgf8 *at the isthmus also resulted in more *Islet1 *expression within the trigeminal placodes (Figure 6A, D; n = 12/12). Similar to *Islet1 *expression, after overexpression of either *Wnt1 *or *Fgf8 *at the isthmus, *Brn3a *expression was more robustly detectable within both lobes of the trigeminal (data not shown). Coronal sections reveal more *Islet1 *positive cells on the injected side (Additional file 3A) and in the case of *Brn3a*, more positive cells can be detected within the ectoderm and delaminating through the mesenchyme (Additional file 3B). The expression of *Islet1 *was also examined using confocal microscopy (Figure 6K, L). The trigeminal lobes on the targeted side appeared more branched and extended (Figure 6L; n = 3/3) compared to controls (Figure 6K), suggesting premature differentiation. Similar to *Pax3 *expression, overexpression of Wnt1 at the midbrain-hindbrain boundary at HH10 resulted in more *Islet1 *expression within the ophthalmic and maxillomandibular branches (Figure 6E, G; n = 18/18). These results confirm that both trigeminal placodes differentiate prematurely after overexpression of *Fgf8*, similar to *Wnt1*. To address if Wnt and FGF signals act synergistically to direct trigeminal development, both constructs were electroporated together into the neural tube at HH10, and *Islet1 *expression was assayed (Figure 6I, J). The expression of both constructs did not further enhance trigeminal placode differentiation over that observed after misexpression of either construct alone (Figure 6J, G compared to Figure 6D). It appears that a threshold of isthmic signals is sufficient to direct trigeminal placode differentiation. In no experimental regime are gross ectopic neurons generated outside the normal placode domains. Similar to the results obtained using neurofilament staining (Figure 5), the precocious expression of neurogenic markers specific to the trigeminal placodes suggests that neuronal differentiation may be occurring prematurely.

**Figure 7:**
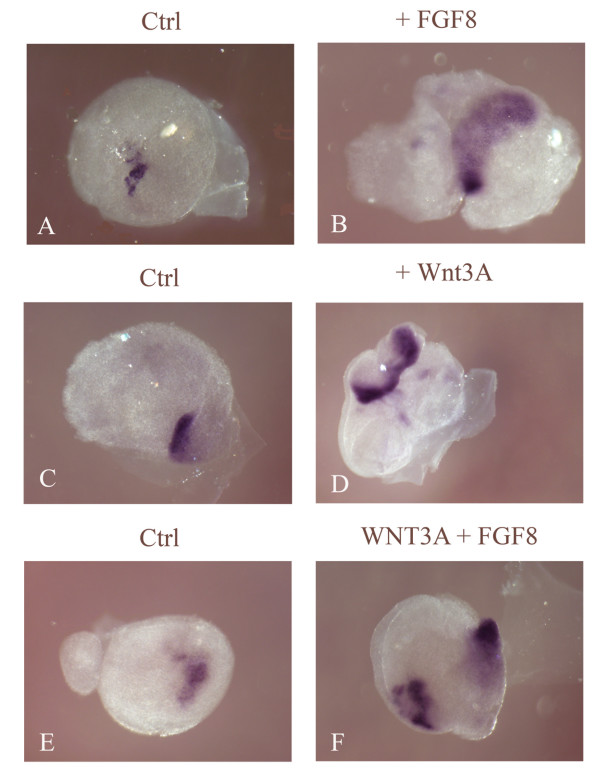
**Addition of fibroblast growth factor (FGF) or Wnt3A results in a similar expression of *Islet1 *in the ectoderm compared to the addition of Wnt3A and FGF8 together.*** In situ *hybridization of *Islet1 *(blue) (A-F) of explants grown over night in culture. Explants in (A, C, E) were left untreated. Treated explants were grown in the presence of FGF8 (B), Wnt3A (D) and Wnt3A and FGF8 (F). FGF8 (B) or Wnt3A (D) addition resulted in a larger domain of *Islet1 *expression compared to controls (A, C). Addition of both Wnt3A and FGF8 (F) did not result in an increase in the domain of *Islet1 *expression compared to either ligand alone.

To further address the question of synergy between Wnt and FGF signals, their activities were tested in cranial ectoderm explant assays. Ectoderm was isolated at HH10 and cultured either alone, or in the presence of FGF8, Wnt3A, or both recombinant proteins for 16 hours. In each condition, a pair of ectoderm explants was isolated from the same embryo. When HH10 ectoderm isolates were cultured in the presence of FGF8 (Figure 7B; n = 8/8) or Wnt3A (Figure 7D; n = 8/8) a larger domain of *Islet1 *expression was detected compared to untreated control explants isolated from the same embryo (Figure 7A, C). Similar to the results obtained *in ovo*, the presence of both Wnt3A and FGF8 in culture did not augment the expression of *Islet1 *(Figure 7F) compared to that observed for either protein alone (Figure 7B, D, F). Therefore, these molecules do not appear to act synergistically, suggesting that a threshold dose of either signaling molecule is sufficient to direct trigeminal placode differentiation.

### FGF signaling via the MAPK pathway directs differentiation of the trigeminal placodes

**Figure 8:**
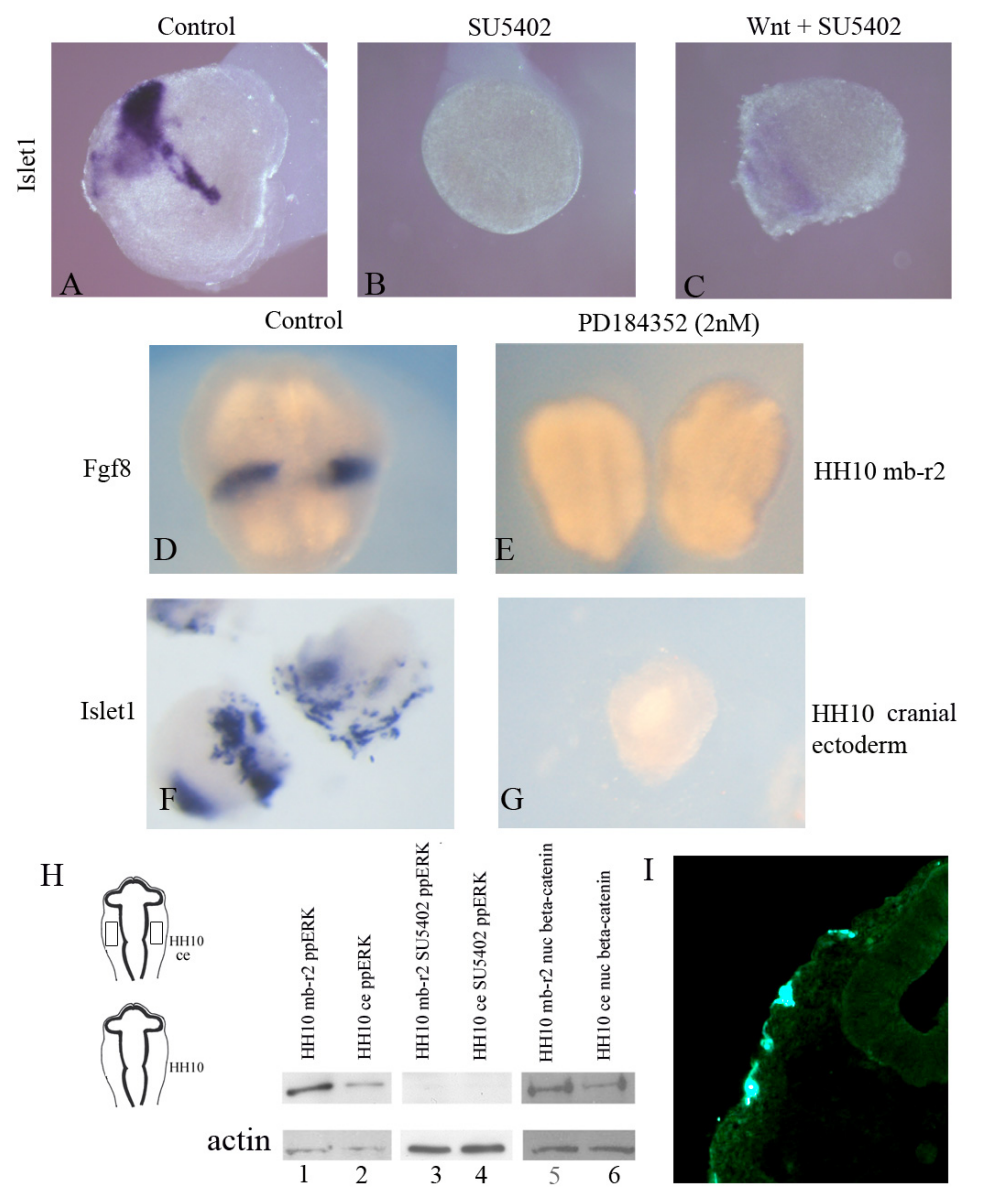
**Fibroblast growth factor (FGF) signaling via the mitogen-activated protein kinase (MAPK) pathway is required for the maintenance of *Islet1 *expression in the trigeminal placodes.**  Cranial ectoderm explants (A-C, F, G) and midbrain to rhombomere 2 (mb-r2) (D, E) were excised at HH10 and grown overnight until approximately HH16. *In situ *hybridizations for *Islet1 *(A-C, F, G) and *Fgf8 *(D-E) expression are shown in blue. Explants were cultured in SU5402 (B), Wnt3A and SU5402 (C) or PD184352 (E, G). The presence of SU5402 resulted in the loss of *Islet1 *expression (B) compared to controls (A). In the presence of SU5402, Wnt3A did not maintain *Islet1 *expression (C). The presence of a selective MEK antagonist (PD184352, 2 nM) led to a loss of *Fgf8 *expression in mb-r2 explants (E) compared to controls (D). Inhibition of MEK activity also resulted in a complete loss of *Islet1 *expression (G) compared to controls (F). (D-G), control explants (left ce) and experimental explants (right ce) were isolated from the same embryo. (H) Western blots of HH10 mb-r2 explants (lanes 1, 3 and 5) and HH10 cranial ectoderm (ce; lanes 2, 4 and 6). Mb-r2 and cranial ectoderm explants lose double phosphorylated ERK1/2 (dpERK1/2) activity in the presence of 50 μM SU5402 (lanes 3 and 4). In all other lanes, isolated tissue is positive for dpErk1/2 at HH10 (lanes 1 and 2), and also for nuclear *β*-catenin (lanes 5 and 6). Actin is shown as a loading control. (I) Immunostain showing the expression of dpERK in the cranial ectoderm.

Electroporation of either Wnt1 or FGF8 at the level of the isthmus expands *Fgf8 *expression (Figure 5 and Additional file 2). Similarly, increasing either Wnt1 or FGF8 levels leads to premature differentiation of the trigeminal placodes (Figures 5 and 6). Wnt signals are, however, dependent on the FGF pathway to initiate the onset of *Pax3 *expression, and subsequent neuronal markers (Figure 4). *In ovo *loss of function analyses suggest that isthmic Wnt signals may not be required after HH10 when the placodal *Pax3 *expression domain is specified (Figure 2). We therefore sought to investigate whether FGF signals themselves may function independently during early neural differentiation of the placodes. When HH10 explants were isolated and cultured alone for 16 hours, they expressed *Islet1 *(Figure 8A; n = 10/10). The presence of SU5402 (50 μM), an FGF receptor antagonist, resulted in a loss of *Islet1 *expression (Figure 8B; n = 10/10). In explants where the FGF pathway was blocked, the addition of Wnt3A was no longer sufficient to maintain *Islet1 *expression (Figure 8C; n = 8/8). These results suggest that the FGF pathway is required to maintain early neuronal differentiation of the trigeminal placodes.

Three well characterized pathways are activated downstream of the FGF receptors, namely the MAPK pathway, the phosphoinositide 3-kinase-AKT and phospholipase Cγ pathways. To test whether MAPK signaling is required for the onset of neuronal differentiation within placodal ectoderm, HH10 explants were treated with a MEK antagonist (PD184352, 5μM) and cultured until they reached approximately HH16. As a control for the activity of the MEK antagonist, HH10 midbrain to rhombomere 2 explants that normally express *Fgf8 *(Figure 8D; n = 4/4) lost this expression when cultured overnight in the presence of PD184352 (Figure 8E). These results are identical to our previous findings using SU5402 [14]. PD184352 was then used to assess whether neuronal differentiation of the trigeminal placodes was dependent on MAPK activity. The presence of PD184352 resulted in a loss of *Islet1 *expression (Figure 8G; n = 12/14) compared to HH10 ectoderm controls (Figure 8F). These results demonstrate that FGF signaling, via the MAPK pathway, is necessary to direct an early differentiation event within the trigeminal placodes. Western blot analysis of midbrain to rhombomere 2 and cranial ectoderm explants at HH10 also detected dpErk, which was downregulated in the presence of either SU5402 (Figure 8H) or PD184352 (a selective MEK antagonist; data not shown). Similar stage explants were also positive for the accumulation of nuclear *β*-catenin. Cryosections of HH15 embryos were also positive for dpERK immunofluorescence within the cranial ectoderm (Figure 8I).

## Discussion

Our aim is to understand the precise origin and identity of the signals and mechanisms that govern the earliest development of the trigeminal placodes and their differentiation into sensory neurons. The trigeminal ganglion develops as a relatively simple two-dimensional structure. First, the placodal precursors marked by *Pax3 *expression are specified in the cranial ectoderm and grow anterior-laterally adjacent to the midbrain-hindbrain boundary and rhombomere 1 [5]. Secondly, these cells begin to delaminate into the underlying mesenchyme in a manner that has recently been described to occur independent of epithelial to mesenchymal transition [17]. Surprisingly, the molecular mechanisms underlying the development of the early ophthalmic and maxillomandibular trigeminal placodes, and further differentiation of the ganglion are poorly understood. A recent finding described how canonical Wnt signaling was involved in the maintenance of cell fate within the developing ophthalmic placode [8]. However, one of the conclusions from this study was that although canonical Wnt signaling was necessary for maintenance of placodal cell fate, it was not sufficient. Here we present data demonstrating that isthmic Wnt signals cooperate with FGF signaling and that, acting together, these are necessary and sufficient for the establishment of the trigeminal placodes. Furthermore, FGF signals maintain differentiation of both ophthalmic and maxillomandibular neurons.

### Isthmic Wnt signals and FGF activity are necessary and sufficient for the formation and differentiation of the trigeminal ganglion

Our observations extend those made by Lassiter *et al*. [8], whereby Wnt signaling within the cranial ectoderm was deemed necessary but not sufficient for the maintenance of placodal cell fate. In their study, overexpression of activated *β*-catenin in the ectoderm itself was not sufficient to promote placodal differentiation. Here, we demonstrate that activation of either Wnt1 or FGF8 signals from within the neural tube is sufficient to direct premature differentiation of the trigeminal placodes and ganglion. In addition, our gain of function studies reveal for the first time that the maxillomandibular lobe of the trigeminal also responds to elevated Wnt and FGF signaling in a manner similar to the ophthalmic branch. It should be noted that while Wnt1 or FGF8 was activated separately within the neural tube *in ovo*, it resulted in the ectopic activation of the other. It was impossible, therefore, to uncouple individual Wnt and FGF ligand activity in this experimental setting. Explants assays were therefore employed to address these issues. We demonstrate that Wnt signals depend on FGF activity to induce the expression of the early ophthalmic placode marker *Pax3*. Loss of function studies show the absolute requirement for neural tube derived Wnt signals in the establishment of early placode development. Additionally, the FGF pathway, via MAPK signaling, is required for the maintenance of early neuronal differentiation. We reveal that Wnt and FGF signals are required to control multiple aspects of trigeminal placode development and early differentiation. Similar requirements for Wnt and FGF signaling have been described for the initiation and maintenance of the otic placodes [18-24]. Together, these findings support that the stereotypical positioning of cranial placodes is directed by localized inductive interactions.

### Wnt and FGF signaling are necessary for early development of the trigeminal placode and FGF signaling controls later maintenance of trigeminal differentiation

Of particular note is the temporal mode in which Wnt and FGF signals function within this system. Both Wnt and FGF signals are required to initiate the onset of *Pax3 *expression in cranial ectoderm explants. Misexpression of Wnt1 within the neural tube also results in an increased number of *Pax3 *positive cells within the ophthalmic placode. Surprisingly, perturbing neural tube derived Wnt signals after HH10 did not affect the development of the ophthalmic placode, despite the fact that we were able to detect a loss of isthmic *Fgf8 *expression as previously described [14]. This result was supported by explant analyses where the presence of a Wnt antagonist after HH12 did not alter *Pax3 *expression. A recent report describes how hindbrain derived Wnt and FGF signals cooperate to regulate otic placode induction in *Xenopus *[18]. In their findings the authors describe how Wnt signals are required early for the induction of *Pax8 *but not for its subsequent maintenance, a function that depends on FGF signaling. An additional report describes how FGF and Wnt signals act together to synergistically promote proliferation while maintaining the cells in an undifferentiated, multipotent state, but act separately to determine cell lineage specification [25]. Thus, it seems that in a given context Wnt and FGF signals can act together to direct a particular cellular response, but may act independently to regulate further downstream events. We demonstrate an earlier than anticipated role for isthmic-derived Wnt signals in regulating the development of the emerging ophthalmic placode but show that this is ultimately dependent on FGF signaling. Subsequently, as early neurons begin to differentiate, Wnt signals are no longer required but FGF activity via the MAPK cascade is essential. These results complement those observations made by Lassiter *et al*. [8], whereby blocking Wnt signaling within the cranial ectoderm led to a loss of *Pax3 *expression, coincident with downstream neuronal differentiation defects. At the time when the *Pax3 *placodal domain is being established, perturbation of neural tube derived Wnt signals greatly reduces the size of the trigeminal placode. This non-cell autonomous effect may act indirectly, and possibly via FGF ligands, to regulate development of the placode within the adjacent ectoderm. Indeed, *Fgf8 *transcripts are reduced in conjunction with a reduction in *Pax3 *placodal expression in loss of function experiments. Similarly, the presence of SFRP2 in midbrain to rhombomere 2 explants diminishes Wnt1 expression, which is itself known to regulate the maintenance of isthmic *Fgf8 *expression. This knock-on effect may result in a mechanism by which both signals maintain differentiation in the ectoderm. Our findings that isthmic Wnt signals are dependent on FGF activity during early placode development may explain some of the differences observed when comparing our analyses with those of Lassiter *et al*. [8]. Perhaps, when Wnt3A is added alone in explant culture, it can act upon additional signaling pathways, such as FGF or platelet-derived growth factor. The latter was recently shown to be involved in placode development [26]. Platelet-derived growth factor ligands alone, however, were not sufficient to induce *Pax3 *expression in pre-placodal ectoderm, implicating the early requirement for additional pathways. An emerging trend is the occurrence of cross-talk between signaling pathways such as Wnt and FGF [27]. Thus, integration of multiple pathways may underlie the complex signaling events that are known to be ongoing in the vicinity of the isthmic organizer.

For the first time, we provide evidence that FGF signaling is necessary to maintain early neuronal differentiation within placodal tissue. In depth analyses have been carried out by a number of groups to study the expression of FGF receptors (FGFR1-4) in the chick brain [5,9,28,29]. Both FGFR2 and FGFR4 are detected within the cranial ectoderm and mesoderm. We demonstrate that MAPK activity is required within this tissue to maintain neurogenic markers, such as Islet1 and Brn3a, and that FGF8b can enhance the expression of neuronal markers. Thus, these results reveal a new role for isthmic FGF signaling in promoting trigeminal placode differentiation.

Previously, FGF signals have been implicated in the early development of the mesencephalic trigeminal nucleus in chick [30]. It is intriguing to consider how many aspects of the trigeminal system are directly or indirectly influenced by Wnt and FGF signaling, whether acting sequentially or simultaneously. The molecular pathway regulating the early development of mesencephalic trigeminal neurons has not yet been dissected, but it is interesting to note that these neurons are born under the influence of isthmic FGF8 signals and lie within a territory at the midline that is also occupied by dorsal *Wnt1 *and *Wnt3A *transcripts [31]. This implies a regulatory mechanism that may be involved in the development of the mesencephalic trigeminal nucleus.

This study describes how Wnt and FGF signals act together to regulate multiple aspects of trigeminal placode development. We demonstrate that Wnt and FGF signals are required for the onset of *Pax3 *expression in the ophthalmic placode. FGF signaling via the MAPK pathway is subsequently involved in the maintenance of early neuronal differentiation events. It will be intriguing to further investigate how Wnt and FGF signaling is integrated in the context of the isthmic organizer to direct common and distinct aspects of trigeminal ganglion differentiation and additionally in the context of the entire trigeminal system.

## Conclusion

Here we describe the isthmic organizer region as a source of secreted factors that control the early development and differentiation of the trigeminal placodes. Isthmic derived Wnt1 cooperates with FGF signals to initiate the onset of the ophthalmic placodal marker *Pax3*. FGF signals are subsequently required to direct the early differentiation events of the ophthalmic and maxillomandibular placodes. The MAPK pathway is essential for the maintenance of *Islet1 *expression, one of the earliest neuronal markers detected within the placodes. In summary, we present for the first time the requirement for isthmic derived Wnt signals and FGF activity to establish and promote differentiation of the trigeminal placodes.

## Abbreviations

dpErk1/2: double phosphorylated Erk1/2; FGF: fibroblast growth factor; FGFR: FGF receptor; GFP: green fluorescent protein; HH: Hamburger and Hamilton stage; MAPK: mitogen-activated protein kinase; PBSTx: phosphate-buffered saline plus 1% Triton X-100, 1% serum; SFRP: secreted frizzled-related protein.

## Competing interests

The authors declare that they have no competing interests.

## Authors' contributions

CAC, LL and SL carried out all experimental procedures. CAC, AG and CMJ conceived the study, evaluated the findings and prepared the manuscript.

## Supplementary Material

Additional file 1 **Wnt promotes crest migration and an increase in *Pax3 *placodal expression.** HH7 embryos were injected with DiI at the neural fold, and subsequently CAGGS Wnt was electroporated into the right side of the neural tube. GFP is observed in green (A), DiI is seen in red (B) and the merged image is shown (C). (D-H) Embryos are stained for *Pax3*. *Pax3 *expression is expanded within the placodal domain (D, F, H, black arrow) compared to the uninjected side (E, G, black arrow). (G, H) Higher magnification images of (D). Of note, the expanded *Pax3 *domain does not overlap with the migrated neural crest (D, black arrow). Click here for file

Additional file 2 **Wnt3A or FGF8 expand both endogenous *Wnt1 *and *Fgf8 *transcripts at the isthmus.** Midbrain to rhombomere 2 explants are shown (A-D) and stained for the expression of FGF8. The presence of either Wnt3A or FGF8 expands the isthmic *Fgf8 *domain (B, D) compared to control explants (A, C). (E-L) Embryos were electroporated with CAGGS FGF8 at HH10 and stained for the expression of Wnt1 (E-G) and FGF8 (I-K). GFP images are shown (H, L). Overexpression of *Fgf8 *results in ectopic *Wnt1 *transcripts (F, G) compared to control and ectopic *Fgf8 *transcripts (J, K) compared to control (I). Click here for file

Additional file 3 Axon outgrowth is enhanced in the presence of Wnt3A and FGF8. HH10 cranial ectoderm explants were dissected and gown on laminin either in the presence of Wnt3A and FGF8 (C, D) or alone (A, B). Axons were stained for the pan-neuronal marker Neurofilament (green). Explants that were grown in the presence of Wnt3A and FGF8 had axons that appeared more bundled (C, D, white arrowheads) than any observed in (A, B). Click here for file
